# The Genetic Basis for Variation in Sensitivity to Lead Toxicity in *Drosophila melanogaster*

**DOI:** 10.1289/ehp.1510513

**Published:** 2016-02-09

**Authors:** Shanshan Zhou, Tatiana V. Morozova, Yasmeen N. Hussain, Sarah E. Luoma, Lenovia McCoy, Akihiko Yamamoto, Trudy F.C. Mackay, Robert R.H. Anholt

**Affiliations:** 1 W.M. Keck Center for Behavioral Biology, Program in Genetics, and; 2 Department of Biological Sciences, North Carolina State University, Raleigh, North Carolina, USA; 3 Department of Biochemistry and Physiology, School of Bioscience and Medicine, Faculty of Health and Medical Sciences, University of Surrey, Guildford, Surrey, United Kingdom

## Abstract

**Background:**

Lead toxicity presents a worldwide health problem, especially due to its adverse effects on cognitive development in children. However, identifying genes that give rise to individual variation in susceptibility to lead toxicity is challenging in human populations.

**Objectives:**

Our goal was to use Drosophila melanogaster to identify evolutionarily conserved candidate genes associated with individual variation in susceptibility to lead exposure.

**Methods:**

To identify candidate genes associated with variation in susceptibility to lead toxicity, we measured effects of lead exposure on development time, viability and adult activity in the Drosophila melanogaster Genetic Reference Panel (DGRP) and performed genome-wide association analyses to identify candidate genes. We used mutants to assess functional causality of candidate genes and constructed a genetic network associated with variation in sensitivity to lead exposure, on which we could superimpose human orthologs.

**Results:**

We found substantial heritabilities for all three traits and identified candidate genes associated with variation in susceptibility to lead exposure for each phenotype. The genetic architectures that determine variation in sensitivity to lead exposure are highly polygenic. Gene ontology and network analyses showed enrichment of genes associated with early development and function of the nervous system.

**Conclusions:**

Drosophila melanogaster presents an advantageous model to study the genetic underpinnings of variation in susceptibility to lead toxicity. Evolutionary conservation of cellular pathways that respond to toxic exposure allows predictions regarding orthologous genes and pathways across phyla. Thus, studies in the D. melanogaster model system can identify candidate susceptibility genes to guide subsequent studies in human populations.

**Citation:**

Zhou S, Morozova TV, Hussain YN, Luoma SE, McCoy L, Yamamoto A, Mackay TF, Anholt RR. 2016. The genetic basis for variation in sensitivity to lead toxicity in Drosophila melanogaster. Environ Health Perspect 124:1062–1070; http://dx.doi.org/10.1289/ehp.1510513

## Introduction

Heavy metal toxicity is a worldwide health problem. Lead exposure, especially, is of concern due to the adverse effects of low concentrations on cognitive development in children (Finkelstein et al. 1998; Solon et al. 2008; Jedrychowski et al. 2009; Mazumdar et al. 2011). Although lead compounds have been phased out of most gasoline products and residential paints, lead exposure in children still remains of concern (Jacobs et al. 2002; Landrigan et al. 2002).

The neurotoxic mechanisms of lead encompass effects on the composition and function of hippocampal NMDA receptors (Neal and Guilarte 2010; Neal et al. 2011) and inhibition of presynaptic calcium channels (He et al. 2009). Previous studies measuring lead blood or bone levels in human populations identified polymorphisms in *ALAD* encoding δ-aminolevulinic acid dehydratase, associated with heme biosynthesis; *VDR*, which encodes the vitamin D receptor; and the hemochromatosis-associated gene *HFE* (Onalaja and Claudio 2000; Warrington et al. 2015; Jhun et al. 2015). In addition, lead-dependent changes in DNA methylation in mice (Sánchez-Martín et al. 2015) have been documented, and Li et al. (2015) identified associations between human lead blood levels in childhood and DNA methylation in adulthood. Despite these advances, however, identifying alleles that may exacerbate or ameliorate exposure risk in human populations remains challenging. Such information is hard to obtain, especially in children, as it is difficult to quantify the onset, duration, and extent of exposure and to measure adverse effects of lead exposure that become manifest at a later stage of development.

Although the effect of genetic background on variation in susceptibility to lead toxicity in human populations is well appreciated (Gundacker et al. 2010; Kim et al. 2014), studies on the genetics of susceptibility to heavy metal toxicity in human populations have been challenging due to the variety of clinical symptoms; uncontrolled environments, often with exposure to multiple toxicants; difficulty in relating phenotypic effect size to toxic dose, especially when symptoms become manifest with a substantial time lag after exposure; uncontrolled genetic backgrounds; and often limited sample sizes for large scale genomic studies. These problems can be alleviated through the identification of candidate risk alleles with human orthologs in model systems.

*Drosophila melanogaster* presents an advantageous model, since the genetic background, environment, and exposure can be controlled precisely. Evolutionary conservation of fundamental cellular pathways that respond to toxic exposure allows us to infer predictions regarding orthologous genes and pathways across phyla (Morozova et al. 2009; Jordan et al. 2012). Thus, studies in the *D. melanogaster* model system can identify candidate susceptibility genes to guide subsequent studies in human populations.

Previous studies identified large genomic regions (quantitative trait loci; QTL) associated with physiological and behavioral responses to lead exposure in recombinant inbred lines derived from Oregon R and Russian 2b parental strains, and documented genome-wide transcript profiles (Ruden et al. 2009). These studies, however, were limited because of restricted genetic diversity contributed by only two parental strains, sparse density of genotypic markers, and lack of replication within lines used for the expression analysis, which precludes statistical assessment of causal lead-induced changes in transcript abundance.

Here, we took advantage of natural genetic and phenotypic variation in a panel of wild-derived inbred lines with fully sequenced and well annotated genomes, the *Drosophila melanogaster* Genetic Reference Panel (DGRP; Mackay et al. 2012; Huang et al. 2014). The DGRP enables genome-wide association (GWA) studies with several advantages. The lines have defined genetic backgrounds; we can control the rearing environment and toxic exposure precisely; and we can quantitatively assess effects of exposure to lead on development time, viability and adult locomotion. Since the lines are inbred, we can conduct repeated measures for toxic exposure on the same genotype. Moreover, we can use mutational analyses to assess whether genes that harbor associated polymorphisms affect the phenotype, thus inferring causality at the gene level.

## Methods

### Fly Stocks and Fitness Traits

We used 200 DGRP lines reared on cornmeal-molasses-yeast medium at 25°C under a 12 hr light-dark cycle. We allowed them to lay eggs on grape juice agar, and collected 50 first instar larvae which we placed either on control medium (2 g of Carolina Formula 4-24® Drosophila medium in 7 mL of water) or medium supplemented with 0.5 mM lead acetate (2 g of Carolina Formula 4-24® Drosophila medium in 7 mL of 0.5 mM lead acetate solution). Grape agar was purchased from Genesee Scientific, Inc. and reconstituted according to the manufacturer’s instructions. In order to promote egg laying, a drop of yeast paste was placed in the center of the grape agar plate. We used five replicate vials per line and medium for measurement of development time and viability. To measure development time we collected and counted eclosing adult flies (i.e. flies emerging from the pupal case) every day and sexed them. We used the mean eclosion day across all flies for each sex as a measurement of development time. Measurements were randomized among replicates and among lines. We used the proportion of surviving adults out of 50 larvae as a measurement of viability. *P{MiET1}* mutants, which are homozygous lines with *Minos* transposon insertions in a common genetic background (Bellen et al. 2011), and their co-isogenic control were obtained from the Bloomington *Drosophila* stock center.

### Activity Analysis

We tested the same individuals for which we assessed development time on lead or control medium for activity. To measure locomotor activity we placed individual flies (20 replicates/sex), either untreated or exposed to lead acetate until eclosion, in tubes containing regular fly food in a Drosophila activity monitoring system (TriKinetics Inc., Waltham, MA), which measures the number of times a given fly crosses an infrared beam (Harbison et al. 2013). Total activity was measured for 2 days.

### Quantitative Genetic Analysis

We partitioned the variance of development time and activity across the two media, using the ANOVA model: *Y* = *μ* + *L* + *S* + *T* + *L × S + L × T + S × T + L × S × T* + ε, where *L* (line) is a random effect, *S* (sex) and *T* (treatment) are fixed effects, and ε is the error variance. Since we did not determine larval sex, we analyzed viability with the ANOVA model *Y* = *μ* + *L* + *T* + *L × T* + ε. We estimated variance components using the restricted maximum likelihood method and calculated broad sense heritability as *H^2^* = σ*^2^_G_*/σ*^2^_P_,* where σ*^2^_G_* is the total genetic variation (σ*^2^_L_* + σ*^2^_L × S_* + σ*^2^_L × T_* + σ*^2^_L × T × S_* for the full model and σ*^2^_L_* + σ*^2^_L × S_* for the analyses within each treatment) and σ*^2^_P_* is the total phenotypic variation, where σ*^2^_P_* = σ*^2^_G_* + ε.

### Genome-Wide Association

We performed GWA analyses for development time, viability and activity on each rearing medium using the pipeline available at the DGRP (http://dgrp2.gnets.ncsu.edu/). The pipeline implements single-variant tests of association for additive effects of variants that are present at minor allele frequencies of at least 0.05. *Wolbachia* infection, a symbiotic bacterium that could change fly physiology and behavior, was found in ~ 53% of the DGRP lines. The effects of *Wolbachia* infection and major genomic inversions were accounted for by including them in the association model in the pipeline (Mackay et al. 2012; Huang et al. 2014). In addition, we performed GWA analysis for sensitivity of development time, viability, and activity, calculated as the difference of line means between flies that were reared on control medium and those that were reared on lead supplemented medium, using the same pipeline. We report the top associations with *p* < 10^–5^, based on quantile-quantile plots, which showed deviations of observed *p-*values from expected values at this threshold. Pairwise linkage disequilibrium was assessed between polymorphic variants using the *r* ^2^ parameterization (Huang et al. 2014) to help evaluate to what extent clustered SNPs segregate independently. For development time and activity, where we have data from both sexes, we performed GWA analyses for females and males separately, averaged between the sexes and for the difference between the sexes.

To further identify alleles associated with differential susceptibility to lead we performed *t*-tests between lead exposed and control flies for each line. We categorized the lines that developed significantly slower on lead medium compared to control medium into “poor performer” and the rest into “good performer” categories for development time. We also categorized lines that have significantly lower viability on lead medium into “poor performers” and the rest into “good performers” for viability. We did a logistic regression on all the top polymorphisms for each trait and sex. The *p*-values for statistical significance after Bonferroni correction are 1.19 × 10^–3^ for viability, 4.42 × 10^–4^ for development time of males, and 6.02 × 10^–4^ for development time of females (α before correction is 0.05).

### Bioinformatics Analyses

We performed gene ontology enrichment analyses with DAVID [Database for Annotation, Visualization and Integrated Discovery (DAVID) Bioinformatics Resources 6.7] (Huang et al. 2009). We annotated DNA variants using the gene models in Flybase release 5.49 (McQuilton et al. 2012). We used the DIOPT–Drosophila RNAi Screening Center (DRSC) Integrative Ortholog Predictive Tool, with all available prediction tools and excluding low score of less than 2, to identify human orthologs (Hu et al. 2011). We mapped genes to genetic interaction databases downloaded from Flybase. We then extracted a network whose edges were either a direct connection between candidate genes or bridged by only one gene not among the candidate gene list. We evaluated the significance of the size of the largest cluster among the subnetworks by a randomization test in which we randomly extracted subnetworks with the same number of input genes. The *p*-value was determined by dividing the number of instances where the size of the largest cluster exceeds the observed largest size by the total number of randomizations (Antonov et al. 2008) (α = 0.05).

### Mutant Analyses

To confirm the GWA results, we tested the top candidate genes using available *P{MiET1}* transposon insertion lines (Bellen et al. 2011). There were 16 mutant lines available to test the top genes with polymorphisms that were associated (*p* < 10^–6^) with variation in development time and 4 to test the top genes with polymorphisms that were associated for activity (*p* < 10^–7^). The *P{MiET1}* transposon insertion lines are homozygous lines that contain single *Minos* transposons in the same genetic background. All mutant lines were tested for development time and activity, as described above, contemporaneously with the transposon-free control. We performed statistical analyses for each mutant line and the control line separately using an ANOVA model of form *Y* = *μ* + *L* + *T* + *L* × *T* + *Rep*(*L* × *T*) + ε to assess the differences between mutant and control lines (*L*) and food treatment (lead and regular food, *T*), where ε is the residual variance. Significance of the Line by Treatment interaction term (*L* × *T*; *p* < 0.05) indicates an effect of the mutation on sensitivity to lead. Sensitivity to lead for development time for tested candidate genes is presented as the difference of mean development time on lead food and regular food between the mutant line and the control line (Pb[Mutant – Control] – Regular food [Mutant – Control]). The data are presented as sensitivity ± SEM, calculated as _√_ *SEl* ^_2_^ + *SEr* ^_2_^, where *l* and *r* are SE for development time on lead and regular food, respectively. The same analysis was performed for activity. We estimated the proportion of candidate genes verified with mutant analysis by combining the success rate in functional validation for males and females in percentages for sexes combined.

## Results

### Dose-Dependent Variation in Sensitivity to Lead Exposure in the DGRP

**Figure 1:**
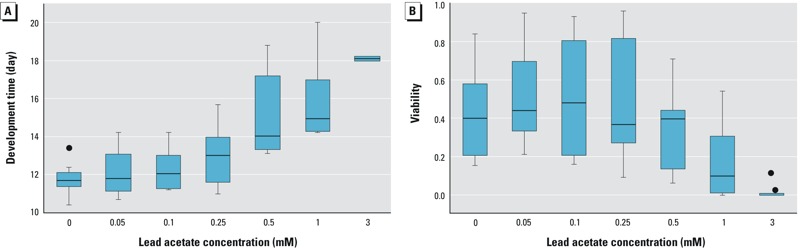
Box plots representing dose–response relationships for flies grown on lead acetate supplemented media for development time (*A*) and viability (*B*) across 10 randomly selected DGRP lines (DGRP_21; DGRP_208; DGRP_304; DGRP_313; DGRP_324; DGRP_335; DGRP_362; DGRP_517; DGRP_732; DGRP_852). Boxes extend from the 25th to the 75th percentile, horizontal bars represent the median, whiskers extend 1.5 times the length of the interquartile range (IQR) above and below the 75th and 25th percentiles, respectively, and outliers are represented as black circles.

Since we expected susceptibility to lead to be dependent on genetic background, we performed dose–response studies on a sample of 10 randomly selected DGRP lines from the total population of 200 lines to establish an optimal concentration for analysis across the DGRP. We reared these lines on media supplemented with concentrations ranging from 0 to 3.0 mM lead acetate from oviposition through eclosion (adult fly emerged), and measured development time and viability (Figure 1). Exposure to lead acetate caused dose-dependent effects both on development time and viability. Development time increased with increasing concentrations of lead, while for viability, variation across lines increased at lower doses of lead, but was followed by a drop in mean viability at higher concentrations. We selected 0.5 mM lead acetate as a discriminating concentration to detect variation for development time and viability across the DGRP, while recognizing that highly sensitive lines may show susceptibility at much lower concentrations.

### Phenotypic Variation in Sensitivity of Development Time and Viability to Lead Exposure in the DGRP

**Figure 2:**
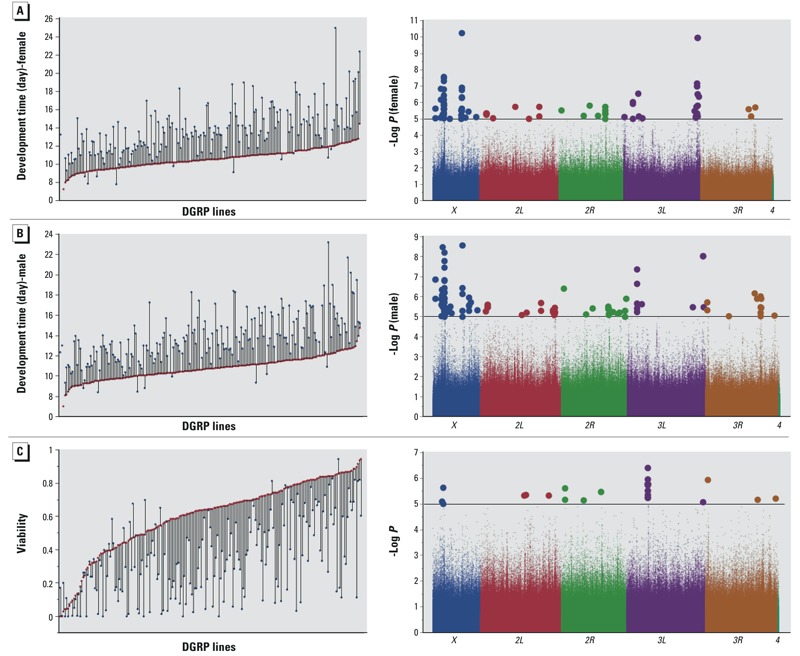
Phenotypic variation (left panels) and genome-wide associations (right panels) for sensitivity to lead exposure for development time of females (*A*) and males (*B*) and viability (*C*). In the left panels, the *x*-axes indicate 200 individual DGRP lines, red symbols correspond to growth on control medium, and blue symbols correspond to growth on medium supplemented with 0.5 mM lead acetate. The differences between the two growth conditions, illustrated by the vertical connecting lines, represent the sensitivity to lead exposure, used for the GWA analyses shown by the Manhattan plots on the right. The chromosome arms are color coded and polymorphic markers above the horizontal line, which designates the *p* < 10^–5^ statistical threshold, are shown as larger circles.

We measured development time and viability of larvae from 200 DGRP lines, which were healthy and easily reared, on standard medium and medium supplemented with 0.5 mM lead acetate. Development time was measured as the number of days it takes for a first instar larva to develop to adult. Mean eclosion time for flies reared on standard medium was 10.7 days with standard deviation of 1.2 days, while lead exposed flies emerged, on average, after 13.7 days with standard deviation of 2.8 days. Mean viability for flies reared on control medium is 60% and 35% on lead supplemented medium. We found extensive variation across the lines for development time and viability both on standard and lead supplemented medium (Figure 2A–C). Whereas some lines appeared marginally or not at all affected by this concentration of lead, 12 of the lines were not viable at this exposure level and 4 of the lines had only male offspring, with extensive variation between these extremes. We excluded both females and males of these 12 lines and females of the 4 lines from GWA analyses for development time. We used the difference between mean phenotypic values of control versus lead exposed flies as a measure of sensitivity to lead exposure. We entered all data in the mixed model ANOVA analysis, while marking development time of non-viable lines on lead medium as missing data (“.”). ANOVA showed significant genotype by environment interaction for both development time (*p* < 0.0001, Table 1) and viability (*p* < 0.0001, Table 2), indicating that sensitivity to lead exposure as measured by development time or viability is strongly dependent on genetic background with estimated heritabilities for both sexes combined of 0.76 and 0.80, respectively. We observed a significant correlation between effects of lead exposure on development time and viability (*R* = 0.449, *p* < 0.0001), so that lines that take longer to develop also tend to have lower viability (see Figure S1). Whereas the majority of the lines perform worse on lead supplemented medium in terms of viability or development time it is of interest to note that a few lines show better survival and faster development on lead supplemented medium than on standard medium (Figure 2A–C). There was no correlation between susceptibility to lead exposure for activity and either viability or development time (data not shown).

**Table 1:** ANOVA for variation in development time of 200 DGRP lines [degrees of freedom (DF); Type III mean squares (MS)]

Analysis	Reduced analyses	Source	DF	MS	Error DF	*F*-Value	*p*-Value	Variance component
Full model		Treatment	1	7012.292	188.68	422.94	< 0.0001	Fixed
		Sex	1	8.690	234.51	9.25	0.003	Fixed
		Sex × treatment	1	5.764	204.10	6.07	0.015	Fixed
		Line	200	47.754	185.92	2.68	< 0.0001	1.728
		Sex × line	199	0.941	185.76	0.99	0.531	0.003
		Line × treatment	187	17.953	187.18	18.87	< 0.0001	1.913
		Sex × line × treatment	183	0.952	2833.00	1.04	0.337	–0.019
		Residual	2,833	0.913				0.920
By treatment	Control	Sex	1	17.451	216.09	97.25	< 0.0001	Fixed
		Line	197	11.241	197.00	64.12	< 0.0001	1.148
		Sex × line	197	0.175	1536.00	0.49	1.000	0.000
		Residual	1,536	0.357				0.337
	Lead	Sex	1	0.104	234.47	0.06	0.802	Fixed
		Line	190	54.177	191.44	32.55	< 0.0001	6.198
		Sex × line	185	1.666	1297.00	1.06	0.287	0.017
		Residual	1,297	1.570				1.573
By treatment and sex	Control female	Line	197	5.568	769.00	15.50	< 0.0001	1.071
		Residual	769	0.359				0.359
	Control male	Line	197	5.963	767.00	16.77	< 0.0001	1.178
		Residual	767	0.356				0.356
	Lead female	Line	185	30.275	634.00	19.84	< 0.0001	6.682
		Residual	634	1.526				1.526
	Lead male	Line	190	26.592	663.00	16.48	< 0.0001	5.797
		Residual	663	1.613				1.614
*h^2^* = (σ*^2^_L_* + σ*^2^_L _*_×_*_ S_* + σ*^2^_L _*_×_*_ T_* + σ*^2^_L _*_×_*_ T _*_×_*_ S_*)/(σ*^2^_L_* + σ*^2^_L _*_×_*_ S_* + σ*^2^_L _*_×_*_ T_* + σ*^2^_L _*_×_*_ T _*_×_*_ S_* + ε) = 0.76.								

**Table 2:** ANOVA for variation in viability 200 DGRP lines [degrees of freedom (DF); Type III mean squares (MS)]

Analysis	Reduced analyses	Source	DF	MS	Error DF	*F*-Value	*p*-Value	Variance component
Full model		Treatment	1	32.139	198.14	271.41	< 0.0001	Fixed
		Line	200	0.398	198.00	3.36	< 0.0001	0.029
		Line × treatment	198	0.119	1597.00	7.52	< 0.0001	0.021
		Residual	1,597	0.016				0.016
By treatment	Control	Line	199	0.286	799.00	15.86	< 0.0001	0.054
		Residual	799	0.018				0.018
	Lead	Line	199	0.237	798.00	17.49	< 0.0001	0.045
		Residual	798	0.014				0.014
*h^2^* = (σ*^2^_L_* + σ*^2^_L _*_×_*_ T_*)/(σ*^2^_L_* + σ*^2^_L _*_×_*_ T_* + ε) = 0.80.								

### Phenotypic Variation in Sensitivity of Activity to Lead Exposure in the DGRP

**Figure 3:**
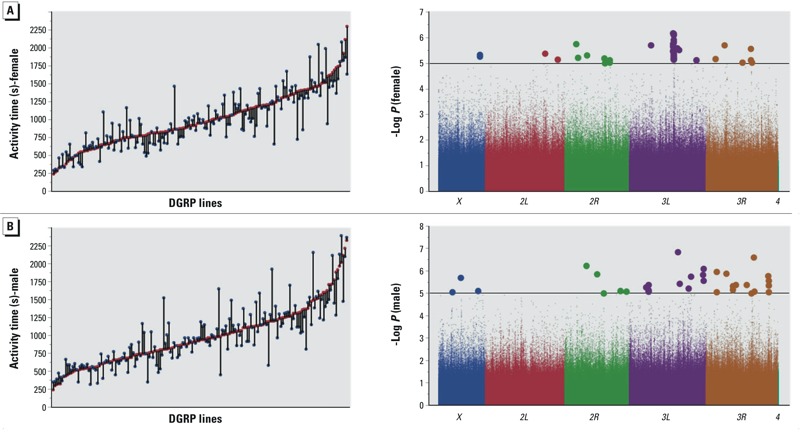
Phenotypic variation (left panels) and genome-wide associations (right panels) for sensitivity to lead exposure for adult locomotor activity of females (*A*) and males (*B*). In the left panels, *x*-axes indicate 166 individual DGRP lines, red symbols correspond to flies grown on control medium, and blue symbols correspond to flies reared on medium supplemented with 0.5 mM lead acetate. The differences between the two growth conditions, illustrated by the vertical connecting lines, represent the sensitivity to lead exposure, used for the GWA analyses shown by the Manhattan plots on the right. The chromosome arms are color coded and polymorphic markers above the horizontal line, which designates the *p* < 10^–5^ statistical threshold, are shown as larger circles.

We analysed adult locomotor activity as a proxy for neural function in 166 DGRP lines that were able to produce enough adult flies when reared on 0.5 mM lead acetate. Single 3–5 day old flies reared on control food or medium supplemented with lead acetate were placed into activity monitor tubes and given regular food, and their activity was measured over 2 days. Total activity was calculated for each individual fly and averaged over 20 male and 20 female flies for each DGRP line and treatment group. Similar to development time and viability, we found extensive variation in locomotion between the lines both reared without and with lead exposure (Figure 3A,B). A significant Line x Treatment term (*p* = 0.0005) indicated substantial genotype by environment interaction (Table 3). Furthermore, some lines exposed to lead show reduced activity, whereas others become hyperactive (Figure 3A,B). The heritability for sexes combined was 0.36.

**Table 3:** ANOVA for adult activity of 166 DGRP lines [degrees of freedom (DF); Type III mean squares (MS)]

Analysis	Reduced analyses	Source	DF	MS	Error DF	*F*-Value	*p*-Value	Variance component
Full model		Treatment	1	739980.1	170.1	0.75	0.3881	Fixed
		Sex	1	275320.6	167.7	0.15	0.6989	Fixed
		Treatment × sex	1	253499.5	173.6	0.43	0.5144	Fixed
		Line	165	20507687.5	176.0	8.76	< 0.0001	121983.4
		Sex × line	165	1923507.8	165	3.14	< 0.0001	18092.6
		Treatment × line	165	1029336.1	165	1.68	0.0005	5230.5
		Treatment × sex × line	165	612337.5	25,600	2.24	< 0.0001	9218.6
		Residual	25,600	273954.7	170.1			273941.6
By treatment	Control	Sex	1	603134.9	167.31	0.43	0.5121	Fixed
		Line	165	11822085.7	165	8.18	< 0.0001	121949.8
		Sex × line	165	1445867.5	13,764	5.98	< 0.0001	27771.5
		Residual	13,764	241671.0				241654.6
	Lead	Sex	1	200.4	176.4	0.00	0.9894	Fixed
		Line	165	10688962.1	165	8.64	< 0.0001	132659.8
		Sex × line	165	1236961.0	11,836	3.97	< 0.0001	26293.8
		Residual	11,836	311497.2	167.3			311485.3
By treatment and sex	Control female	Line	165	6368290.1	6,874	27.93	< 0.0001	141348.8
		Residual	6,874	228037.9				228030.5
	Control male	Line	165	6941945.0	6,890	27.19	< 0.0001	157929.9
		Residual	6,890	255272.5				255270.7
	Lead female	Line	165	5769243.4	5,884	25.72	< 0.0001	146230.3
		Residual	5,884	224273.1				224276.7
	Lead male	Line	165	6668344.8	5,952	16.77	< 0.0001	168890.3
		Residual	5,952	397724.8	6,874			397735.5
Twenty-four lines were excluded from this analysis because of low yield of adult flies. *h^2^* = (σ*^2^_L_* + σ*^2^_L __×__ S_* + σ*^2^_L _*_×_*_ T_* + σ*^2^_L _*_×_*_ T _*_×_*_ S_*)/(σ*^2^_L_* + σ*^2^_L _*_×_*_ S_* + σ*^2^_L _*_×_*_ T_* + σ*^2^_L _*_×_*_ T _*_×_*_ S_* + ε) = 0.36.								

In summary, we observed significant differences in the effects of exposure to lead acetate on development, with 12 of 200 lines failing to eclose on both sexes and 4 lines had no viable female when developed on media with 0.5 mM lead acetate. In flies that survived to adulthood, developmental exposure to lead acetate caused both decreased and increased locomotion compared with controls, depending on the genotype.

### Identification of Candidate Genes Associated with Variation in Lead Sensitivity for Development Time and Viability

The high heritabilities for sensitivity of development time and viability to lead exposure indicate a substantial genetic contribution to the phenotypic variation, which provides a favorable scenario for GWA analyses. Quantile-quantile plots indicated deviations of observed versus expected *p*-values at *p* < 10^–5^ (see Figure S2). Therefore, we report results of single marker-based GWA analyses for all associations with *p* < 10^–5^ in both sexes combined (for viability), or in males or females or both sexes combined (for development). With this reporting threshold we identified a total of 216 polymorphisms associated with variation in the effect of lead on development time from all GWA analyses combining data from GWA for sexes separately, for averages between the sexes, and for differences between the sexes. These polymorphisms include 159 in or near 123 genes, and 57 in 25 intergenic regions (see Excel File S1). We looked at genes that are within 1 kb from a significant polymorphism; thus, a single polymorphism can be associated with multiple genes, and, *vice versa*, single genes may be associated with multiple polymorphisms. When we analyzed associations separately for males and females, we identified 113 significant polymorphisms (*p* < 10^–5^) for males (81 in or near 45 genes, and 32 in 12 intergenic regions) and 84 for females (65 in or near 48 genes, and 19 in 8 intergenic regions) (Figure 2A,B; see also Excel File S1). Similarly, we identified 42 polymorphisms associated with variation in the effect of lead on viability, including 34 in or near 26 genes and 8 in 7 intergenic regions (Figure 2C; see also Excel File S2). Several genes contain at least 4 SNPs associated with variation in sensitivity of development time from analyses for sexes separately and sexes combined, including *dpr8*, *CG13954*, *CG42673*, *NetB*, *drd*, *Pdfr*, *Fs(1)Yb* and *SPR* (see Excel File S1). In addition, *bbg* contains 16 intronic SNPs associated with variation in sensitivity of viability to lead exposure (see Excel File S2). Despite the large number of SNPs associated with single genes, they are not in complete linkage disequilibrium (data not shown). Except for *dpr8*, we find no overlap between candidate genes associated with variation in development time and viability, which indicates that these phenotypes are affected by different aspects of the genetic architecture associated with lead sensitivity. Flybase annotations (http://www.flybase.org) of genes associated with variation in sensitivity to lead implicate several genes that are associated with early development, particularly development and function of the nervous system, such as neuropeptide signaling, neurogenesis, axonogenesis, axon guidance, Notch signaling, sensory organ development, cell adhesion, glial cell differentiation, and neurotransmitter signaling (see Excel Files S3 and S4).

Most DGRP lines develop faster during the larval stage and have higher viability to adulthood on control food than food supplemented with lead acetate. However, females from 22 lines and males from 21 lines (both sexes in 16 lines) developed statistically at the same rate or faster on lead food and 76 lines are no different from or are more viable on lead supplemented food compared to the control. These DGRP lines were designated as “good performers” and lines that performed significantly worse on lead-supplemented food as “poor performers” (see Excel File S5). We then did logistic regressions for sexes separately on the 216 polymorphisms with *p* < 10^–5^, which we identified previously from the GWA analyses for sensitivity of development time for sexes separately and sexes combined. Similarly, we did logistic regression for sexes combined on the 42 polymorphisms with *p* < 10^–5^ which we identified previously from the GWA analyses for sensitivity of viability. After Bonferroni correction of *p*-values, we obtained a subset of genes with polymorphisms that are enriched among good performing lines, (i.e., alleles that are protective against lead exposure; see Excel File S6). For development time, we found 46 SNPs and 1 deletion polymorphism in or near 26 genes and five intergenic regions for females and 50 SNPs and four indels in or near 16 genes and six intergenic regions for males (see Excel File S6). Among them, 10 genes were in common between females and males including *drop dead*, which affects digestion and response to salt stress, *Insulin-like peptide 2* and *Insulin-like peptide 3* (tagged by the same SNP), *Evi5,* which regulates GTPase activity, and *female sterile (1) Yb,* which affects both germ-line and somatic stem cell division. In addition, SNPs in one long non-coding RNA, *CR44190*, and downstream of *mir-982* are enriched in good performing females. For viability, we found 13 SNPs and 1 deletion polymorphism in or near 14 genes and four intergenic regions (see Excel File S6), including several genes involved in development, neurogenesis, neurotransmitter activity and an immune response gene (see Excel File S4).

### Identification of Candidate Genes Associated with Variation in Lead Sensitivity for Activity

GWA analysis for variation in sensitivity to lead exposure for activity revealed 114 polymorphisms in or near 68 genes and 12 intergenic regions when sexes were combined in the analysis (see Excel File S7). Gene ontology analysis showed significant enrichment for axon guidance, neuron differentiation, regulation of transcription and cell morphogenesis (see Excel File S8). We identified 33 polymorphisms in or near 21 genes and in 7 intergenic regions when males were analyzed separately (Figure 3B; see also Excel File S7), and 42 polymorphisms in or near 23 genes when females were analyzed separately (Figure 3A; see also Excel File S7). Interestingly, for females 21 SNPs (half of the polymorphisms) were located in *Snap25* that encodes a gene product associated with synaptic transmission.

The phenotypic correlation between males and females of sensitivity of activity was small, but significant (*R* = 0.26, *p* = 0.004); however, there was no overlap at the polymorphism or gene levels between males and females, indicating that the genetic architectures that determine variation in sensitivity to lead exposure in terms of adult activity are distinct for males and females. Furthermore, only *Ptp99A* was in common between sensitivity for development time and activity, but different SNPs were associated with each phenotype. There was no overlap between genes and polymorphisms associated with variation in sensitivity to lead for viability and activity.

### Mutational Analysis of Candidate Genes to Infer Causal Associations with Sensitivity to Lead Exposure

**Figure 4:**
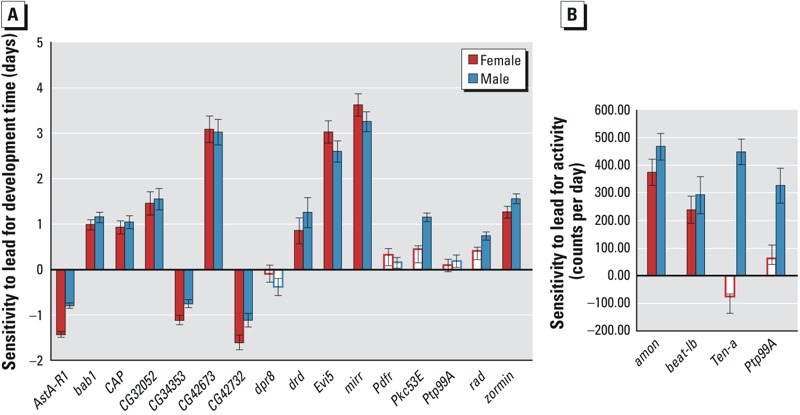
Mutational analysis of candidate genes associated with variation in sensitivity to lead exposure for development time (*A*) and activity (*B*). The bar graphs represent differences between mutants grown on lead and control medium minus the differences of co-isogenic control flies grown on the corresponding media. Red bars indicate females and blue bars indicate males that show significant differences between the mutants and the control as indicated by a statistically significant Line by Treatment interaction term (*p* < 0.05) in an ANOVA analysis of form *Y* = *μ* + *L* + T + *L *×* T* + ε, where *L* designates mutant and control, *T* indicates treatment and ε the residual error variance. White bars indicate no significant differences. Values in panel A are derived from 5 replicates with 50 larvae per replicate and in panel B from 30 flies per sex.

To evaluate functional causality we asked to what extent candidate genes (*p* < 10^–5^) that harbor associated polymorphisms themselves contribute to sensitivity to lead exposure. We selected 16 available co-isogenic *P{MiET1}* mutants from the Bloomington Stock center (Bellen et al. 2011) and compared differences in development time between the mutants grown on control and lead supplemented medium with the difference of the co-isogenic control reared on the same media (Figure 4A). We found that 11 females and 13 males of *P{MiET1}* mutants showed significant differences in sensitivity to lead exposure from the control (*p* < 0.05). We estimated the proportion of candidate genes of which mutants affected sensitivity of development time to lead exposure as 75% by averaging the success rate for males and females as follows: ((11/16) + (13/16))/2 × 100%. It is of interest to note that insertion of the *P{MiET1}* transposon in *AstA-R1*, *CG34353* and *CG42732* provides protection against lead exposure with mutant lines developing faster on lead than the control. *AstA-R1* encodes the Allatostatin A receptor 1, a G protein-coupled receptor that mediates neuropeptide signaling (Larsen et al. 2001), *CG34353* encodes an immunoglobulin-domain containing protein of unknown function, and *CG42732* encodes a potassium channel implicated with neurogenesis (Neumüller et al. 2011).

We also tested available mutants of four of the top candidate genes (*p* < 10^–5^) associated with the effect of lead exposure on variation in adult activity (*amon*, *beat-Ib*, *Ten-A*, *Ptp99A*; Figure 4B). Compared to the control, all of the mutants were more active after lead exposure in males, and *amon* and *beat-Ib* also affected activity after lead exposure in females. (*p* < 0.05; [(4/4) +(2/4)]/2 × 100% = 75%).

### A Network of Genes Associated with Variation in Lead Sensitivity

**Figure 5:**
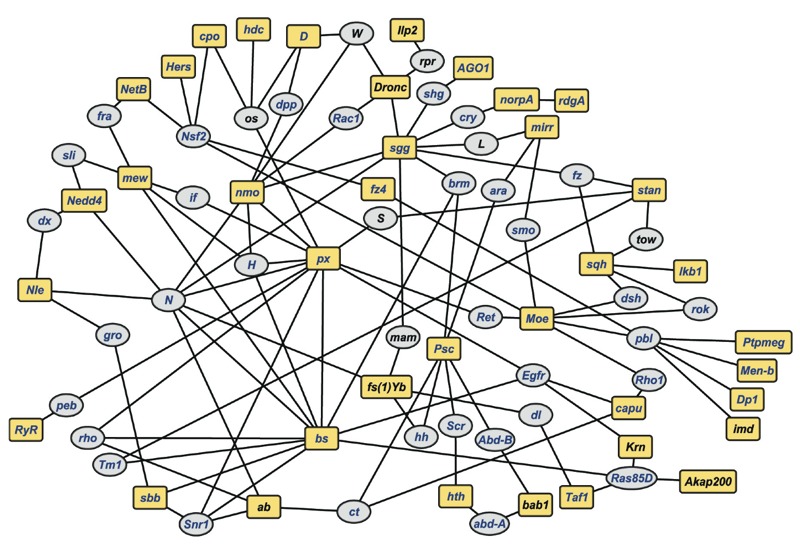
A genetic network for susceptibility to lead exposure. The network was derived from candidate genes identified in GWA analyses for development time, viability and activity (see Excel Files S1, S2, S7). Yellow square boxes indicate candidate genes associated with any of these traits, while gray ovals represent computationally recruited intermediate genes. Blue font indicates genes with human orthologs, identified with the DRSC Integrative Ortholog Prediction Tool (Hu et al. 2011). Drosophila gene annotations are based on Flybase, version 5.49 (http://www.flybase.org). See also Excel File S9 for detailed connections between genes in the network.

Our observations reveal a highly polygenic genetic architecture that underlies variation in sensitivity to lead toxicity. To assess global connectivity between candidate genes, we performed network analysis with 215 unique genes identified from all the GWA analyses for both sexes separately and sexes combined for all three traits (see Excel Files S1, S2, S7). These genes are pleiotropic and for many of them genetic interactions have been documented in Flybase (http://www.flybase.org; McQuilton et al. 2012). We used this information to construct a computationally predicted network of genetically interacting genes, allowing one missing gene (i.e*.,* a gene connecting two candidate genes), which itself did not harbor a variant associated with phenotypic variation (Antonov et al. 2008). This analysis revealed a network of 38 candidate genes from the GWA analyses and 40 computationally recruited intermediate genes (Figure 5). The probability that this network would have arisen when the same number of genes are sampled at random is *p* = 0.025. Among these genes 64 (82%) have human orthologs, thus, enabling us to superimpose a candidate network of human orthologs on their Drosophila counterparts (Figure 5). Gene ontology enrichment analysis of the genes in the network shows significant enrichment for genes associated with early development, including neuron development (*p* = 3.6 × 10^–13^; see Excel File S9). Thus, variation in sensitivity to lead toxicity may result from subtle variations in neuronal connectivity during early development.

## Discussion

Although the clinical effects and pharmacodynamics of heavy metal toxicity have been extensively studied, relatively little is known about the genetic factors that determine individual variation in sensitivity to toxic heavy metal exposure. A few human studies have examined associations of polymorphisms in candidate genes with blood or bone lead concentrations (Tekin et al. 2012; Whitfield et al. 2007; Warrington et al. 2015; Jhun et al. 2015) and with maternal lead burden and infant birth weight (Cantonwine et al. 2010), but genetic studies on human populations have often been inconclusive, mostly due to limited statistical power (reviewed in Gundacker et al. 2010). We took advantage of natural variants segregating in the DGRP to identify DNA sequence variants associated with susceptibility to lead toxicity. The analysis of natural variants has advantages compared to conventional mutagenesis screens since null mutations in developmental genes are often homozygous lethal and segregating variation in the DGRP is expected to more closely mimic genetic variation in human populations (Mackay et al. 2012; Huang et al. 2014).

Previous studies have documented delayed development as a result of exposure to lead acetate in Drosophila (Cohn et al. 1992; Akins et al. 1992; Hirsch et al. 2009). We observed extensive variation in sensitivity to lead exposure for development time, viability, and adult activity. Whereas some lines appeared unaffected by exposure to lead acetate, 24 DGRP lines did not develop at all on lead-supplemented medium. These lines are of interest for a case–control study, but the currently available sample size is too limited to provide sufficient power for such an analysis. Similarly, the differences between “good” and “poor” performers are likely due to gene–gene interactions that are either deleterious or protective upon exposure to lead depending on the genetic context.

We observed little overlap among SNPs that are associated with variation in development time under control and treatment conditions (a SNP in *CG43672* and an intergenic SNP) (data not shown). However, a substantial fraction, 34 genes, associated with variation in development time *per se* on lead supplemented medium is also associated with variation in sensitivity in development time to lead exposure. (i.e., the difference between growth on lead acetate supplemented medium and standard food medium). This represents an example of gene by environment interaction, (i.e., variation in sensitivity to lead recruits a suite of allelic variants that is distinct from those that contribute variation in development time under standard growth conditions). Further evidence for extensive genotype by environment interaction comes from the effects of previous lead exposure on adult locomotor activity, which in some genetic backgrounds is reduced and in others manifests as hyperactivity. Since previous studies have shown that epistatic interactions are an important feature of the genetic architecture of complex traits (Huang et al. 2012; Swarup et al. 2013), we speculate that gene–gene interactions are likely to contribute to the manifestation of genotype by environment effects for sensitivity to lead exposure.

The *p*-value we used to declare significance does not meet a Bonferroni correction for multiple testing. We performed association tests using ~ 2 million common (MAF > 0.05) variants, which would translate to a Bonferroni-corrected significance threshold of ~ 2.5 × 10^–8^. Only variants with very large effects can be detected at this significance level with a sample of 200 lines. However, unlike for humans, we cannot readily increase the sample size without generating and sequencing more inbred lines, which takes many years. Therefore, we sought evidence for a statistical signal by examining the quantile-quantile plots (see Figure S2), which show a departure from random expectation below *p* < 10^–5^. An advantage of the Drosophila model is the ability to use co-isogenic hypomorphic *P{MiET1}* mutants to assess the phenotypic effects of candidate genes harboring sequence variants associated with phenotypic variation in the DGRP. As indicated in the Results, we observed that 75% of tested mutants corresponding to candidate genes affected sensitivity to lead exposure averaged over both sexes. This percentage is similar to functional validation rates obtained with previous GWA studies on the DGRP (Jordan et al. 2012; Weber et al. 2012; Harbison et al. 2013; Swarup et al. 2013; Arya et al. 2015).

We combined candidate genes from all analyses to obtain sufficient statistical power for gene ontology enrichment and network analyses. These analyses showed a genetic network with significant enrichment of early developmental genes, including genes associated with development of the nervous system. This observation is consistent with a previous study, which identified enrichment of neurodevelopmental genes from an analysis of transcriptional profiles among 75 recombinant inbred lines grown either on control medium or medium supplemented with lead acetate (Hirsch et al. 2009). We note that the network we identified is a consensus network and that phenotypic effect sizes of its constituent genes and their connections are likely to change dynamically under different growth conditions in a sex-dependent manner. We also note that the current study does not provide information about the mechanism(s) by which exposure to lead results in cytotoxic injury. The genes we identify and the network that emerged do not *a priori* provide direct targets for interactions with the heavy metal. Rather, they specify a genomic blueprint that dictates subtle developmental variations that culminate in variation in the cellular response to toxic injury. It is of interest to note that many of the genes we identified from our GWA studies have human orthologs (see Excel Files S7 and S8), which may guide future studies on variation in individual susceptibility to lead neurotoxicity in human populations.

## Conclusions

Our GWA analyses reveal a highly polygenic genetic architecture that underlies variation in sensitivity to lead toxicity in the DGRP, which may give rise to subtle variations in neuronal connectivity during early development. Candidate genes we identified include genes with human orthologs, thus providing a genetic framework that can guide future studies in human populations.

## Supplemental Material

(298 KB) PDF

(280 KB) ZIP
