# Supplementary material for: The Genetic Basis for Variation in Sensitivity to Lead Toxicity in Drosophila melanogaster
Source: Environ Health Perspect. 2016 Feb 9;124(7):1062–70. doi: 10.1289/ehp.1510513 (PMC4937873; doi:10.1289/ehp.1510513)
Supplement: (298 KB) PDF [file ehp.1510513.s001.acco.pdf]

*Environ Health Perspect*
DOI: 10.1289/ehp.1510513

**Note to readers with disabilities:** *EHP* strives to ensure that all journal content is accessible to all readers. However, some figures and Supplemental Material published in *EHP* articles may not conform to 508 standards due to the complexity of the information being presented. If you need assistance accessing journal content, please contact ehp508@niehs.nih.gov. Our staff will work with you to assess and meet your accessibility needs within 3 working days.

# Supplemental Material

# The Genetic Basis for Variation in Sensitivity to Lead Toxicity in *Drosophila melanogaster*

Shanshan Zhou, Tatiana V. Morozova, Yasmeen N. Hussain, Sarah E. Luoma, Lenovia McCoy, Akihiko Yamamoto, Trudy F.C. Mackay, and Robert R.H. Anholt

## Table of Contents

**Figure S1**. Correlation diagram between sensitivities of development time and viability to lead exposure among DGRP lines.

**Figure S2**. Quantile-quantile plots for development time and viability, vertical line indicates *P*-value = $10^{-5}$.

**Additional files**

**Supplemental Code and Data Zip File**

**Supplemental Code and Data Index**

**Excel File S1.** GWA analysis for development time.

**Excel File S2.** GWA analysis for viability.

**Excel File S3.** Flybase GO categories for genes associated with variation in development time in the DGRP.

**Excel File S4.** Flybase GO categories for genes associated with variation in viability in the DGRP.

**Excel File S5.** Performer groups for logistic regression analysis.

**Excel File S6.** Genes with protective alleles enriched in “good performer” lines identified through logistic regression.

**Excel File S7.** GWA analysis for adult activity.

**Excel File S8.** Flybase GO categories for genes associated with variation in activity in the DGRP.

**Excel File S9.** GO enrichment analysis using default DAVID settings at level 4.

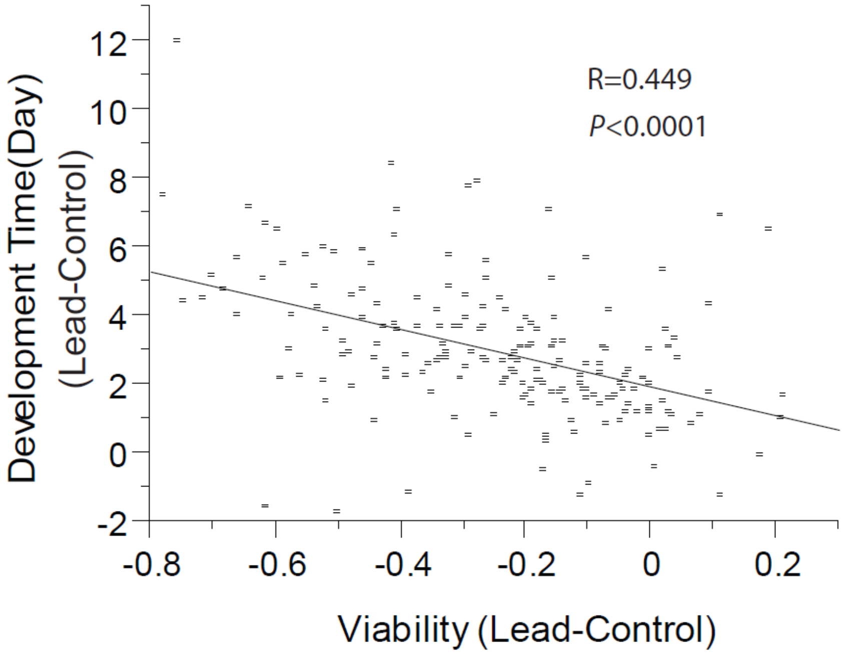


Figure S1 - Correlation diagram between sensitivities of development time and viability to lead exposure among DGRP lines.

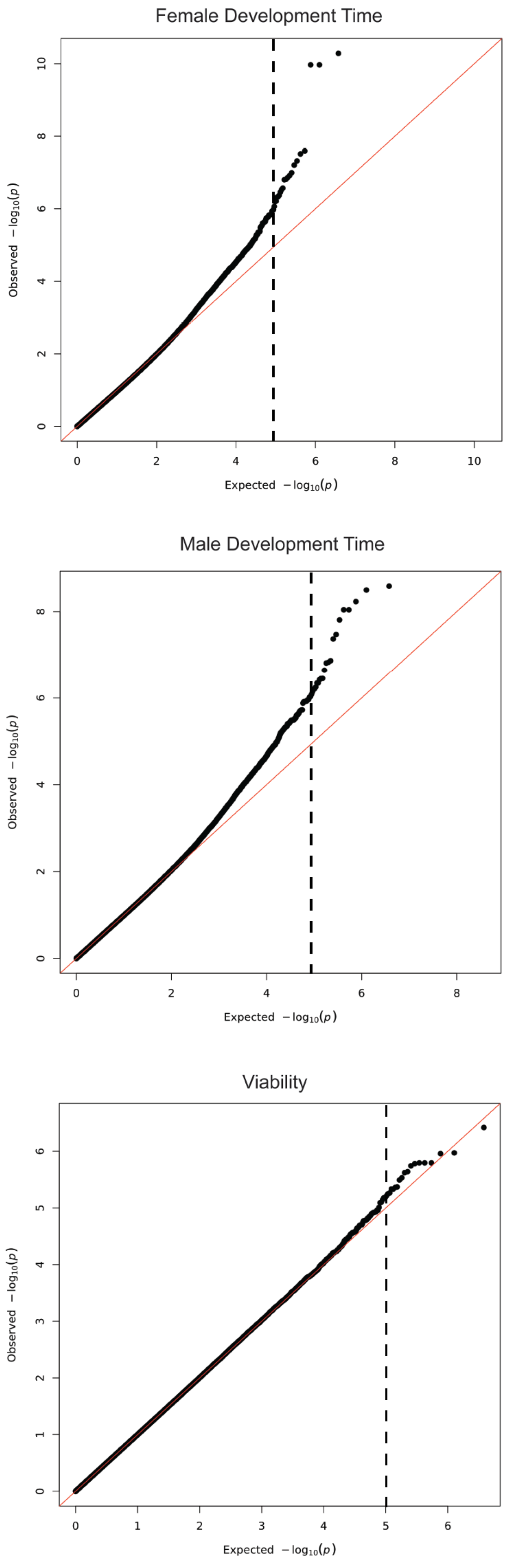


Figure S2 - Quantile-quantile plots for development time and viability, vertical line indicates *P*-value = $10^{-5}$.
